# In Vitro, In Silico, and In Vivo Evaluation of Antiplasmodial Activity of Ursodeoxycholic Acid Following GNPS Dereplication of an Active *Streptomyces* sp. Fraction

**DOI:** 10.3390/ph19060958

**Published:** 2026-06-20

**Authors:** Nanang R. Ariefta, Baldorj Pagmadulam, Takako Aboshi, Yoshifumi Nishikawa

**Affiliations:** 1 National Research Center for Protozoan Diseases, Obihiro University of Agriculture and Veterinary Medicine, Inadacho, Obihiro 080-8555, Japan; nanang.ariefta@gmail.com (N.R.A.); pagmadulam1028@gmail.com (B.P.); 2 Laboratory of Microbial Synthesis, Institute of Biology, Mongolian Academy of Sciences, Bayanzurkh District, 12 Khoroo, Ulaanbaatar 13270, Mongolia; 3 Department of Life, Food, and Environmental Sciences, Faculty of Agriculture, Yamagata University, Wakaba-machi 1-23, Tsuruoka 997-8555, Japan; taboshi@tds1.tr.yamagata-u.ac.jp

**Keywords:** *Streptomyces*, Mongolian, ursodeoxycholic acid, antiplasmodial, drug resistant, *Plasmodium falciparum*, *Plasmodium yoelii*

## Abstract

**Background/Objectives:** The emergence of drug-resistant *Plasmodium falciparum* highlights the need for new antiplasmodial compounds with distinct mechanisms of action. Microbial secondary metabolites, particularly from *Streptomyces* species, remain important sources of bioactive molecules. This study aimed to evaluate antiplasmodial metabolites associated with a Mongolian *Streptomyces* isolate. **Methods:** *Streptomyces* sp. strain D10 was isolated from Mongolian soil samples and extracted with ethyl acetate. Bioassay-guided fractionation was performed, followed by LC–HRMS analysis and GNPS-based spectral dereplication. Antiplasmodial activity was evaluated against *P. falciparum* 3D7, K1, and Dd2 strains using a SYBR Green I assay. Cytotoxicity was assessed in HSF cells. Stage-specific susceptibility assays were conducted using synchronized 3D7 parasites. Comparative docking analyses against β-hematin and the chloroquine resistance transporter (PfCRT), together with target prediction and molecular docking analyses, were performed to explore potential mechanisms. In vivo efficacy was evaluated using a *Plasmodium yoelii* 17XNL mouse model. **Results:** Fractionation yielded an active fraction (C2), and LC–HRMS and GNPS-based dereplication suggested a bile acid-like metabolite, with ursodeoxycholic acid (UDCA) returned as a putative spectral library candidate associated with fraction C2. Fraction C2 and UDCA showed comparable antiplasmodial activity against *P. falciparum* 3D7 (IC_50_ = 6.55 ± 3.00 and 4.68 ± 0. 65 µg/mL, respectively) without detectable cytotoxicity up to 200 µg/mL. Activity was retained against multidrug-resistant K1 and Dd2 strains. Stage-specific assays demonstrated inhibitory activity across ring, trophozoite, and schizont stages without significant stage-dependent differences. Comparative docking analyses suggested interaction profiles distinct from chloroquine in β-hematin and PfCRT models. Additional docking analyses identified PfGluPho, PfMAPK, and PfPFT-β as potential targets. In vivo, UDCA reduced parasitemia in a dose-dependent manner without significant toxicity. **Conclusions:** UDCA exhibited moderate antiplasmodial activity across in vitro, in silico, and in vivo evaluations with a favorable selectivity profile, supporting further investigation of bile acid-like metabolites as potential antimalarial scaffolds.

## 1. Introduction

Malaria remains a major global health problem, particularly due to the emergence and spread of drug-resistant *Plasmodium falciparum* strains [1]. The reduced effectiveness of current antimalarial drugs, including reports of delayed artemisinin response, highlights the need for new compounds with different structures and modes of action. Natural products continue to be an important source of antiparasitic agents, especially those derived from microorganisms [2]. Among these, actinomycetes, particularly *Streptomyces* species, are well known for producing a wide range of bioactive secondary metabolites [3]. Soil-derived *Streptomyces* from less-explored environments are considered promising sources of new compounds [4]. In this context, Mongolian soils have been reported to contain diverse actinomycetes capable of producing structurally vast bioactive metabolites, as demonstrated in previous studies [5,6,7]. However, the chemical characterization of these metabolites and their relevance to antimalarial drug discovery remain limited. In addition, recent studies have highlighted the potential of multi-target or phenotypic approaches in antimalarial drug discovery, as single-target drugs are more prone to resistance development [8,9]. Compounds with broader biological activities, including those affecting redox balance [10], membrane integrity [11], and signaling pathways [11,12], may provide alternative strategies for parasite control.

In this study, *Streptomyces* strains were isolated from soil samples collected in Mongolia and cultivated under standard fermentation conditions. The resulting crude extracts were fractionated and analyzed using LC–HRMS to identify potential bioactive compounds. Among the detected metabolites, Global Natural Products Social Molecular Networking (GNPS)-based spectral dereplication [13] yielded a tentative bile acid-related annotation, with UDCA returned as the closest spectral library match associated with the active fraction. The antiplasmodial activity of the extracts and a commercial UDCA standard, selected based on this annotation, was evaluated against *P. falciparum* strains, including drug-sensitive (3D7) and multidrug-resistant strains (K1 and Dd2), using a SYBR Green I-based assay. Cytotoxicity was assessed in mammalian cell lines (HSF cells) to determine selectivity, and in vivo efficacy was evaluated using a *P. yoelii* 17XNL mouse model. In addition, comparative target simulation, target prediction, and molecular docking were performed to explore potential mechanisms. The results demonstrated that standard UDCA exhibits moderate antiplasmodial activity in vitro and in vivo and suggest a possible multi-target mode of action. These findings support the potential of microbial-derived bile acid–like compounds as candidates for further investigation in antimalarial drug discovery.

## 2. Results

### 2.1. Isolation and Identification of Actinomycetes

In this study, an actinomycete strain (D10) was isolated from soil samples collected in Selenge Province, Mongolia. Colonies were obtained on agar media following incubation at 28 °C for 7–14 days. Colonies exhibiting typical actinomycete morphology were selected and purified through repeated streaking. Genomic DNA was extracted, and the 16S rRNA gene was sequenced using a 3130xl Genetic Analyzer. BLAST (v.2.17.0) analysis against the NCBI database showed that the strain shared 93.5% sequence identity with its closest relative, *Streptomyces* sp. This relatively low sequence similarity suggests that the isolate may represent a phylogenetically distinct *Streptomyces*-related strain (Supplementary Figure S1). Therefore, additional taxonomic analyses, including extended 16S rRNA gene sequence comparison [14], multi-locus sequence analysis (MLSA) [15], and comprehensive phylogenetic characterization, will be necessary to resolve its species-level classification.

### 2.2. In Vitro Antiplasmodial Activities of Crude Extract and Fraction from Streptomyces sp. Strain D10

**Figure 1:**
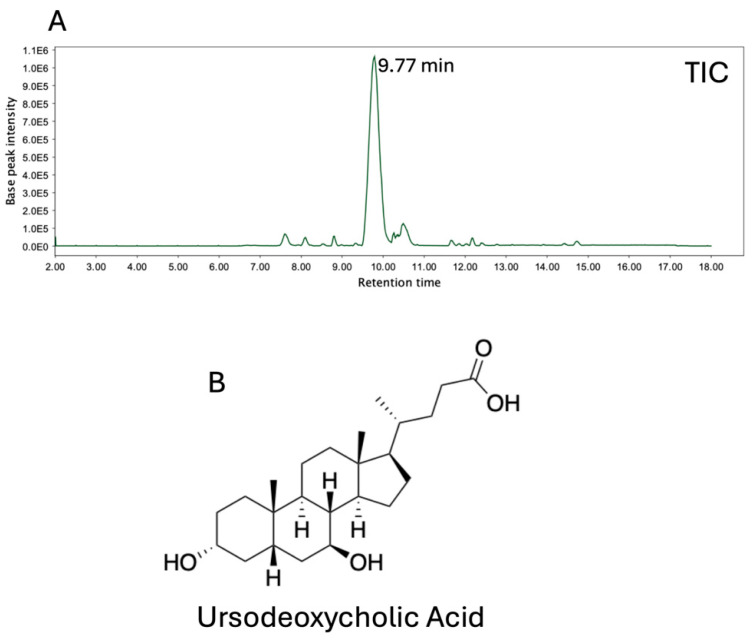
LC–HRMS analysis of the active fraction C2 and candidate compound selected for biological evaluation. (**A**) Total ion chromatogram (TIC) of fraction C2 showing a dominant peak at RT 9.77 min. (**B**) Chemical structure of commercial ursodeoxycholic acid (UDCA), which was selected for subsequent biological evaluation based on GNPS spectral dereplication results.

The crude extract of *Streptomyces* sp. D10 showed inhibitory activity against *P. falciparum* 3D7, with 82.3% inhibition at 100 µg/mL. Following chromatographic fractionation, two major fractions (C1 and C2) were obtained. Fraction C1 exhibited moderate antiplasmodial activity with an IC_50_ value of 23.80 µg/mL against *P. falciparum* 3D7, whereas fraction C2 showed higher potency with an IC_50_ value of 6.55 µg/mL (Supplementary Figures S2 and S3). LC–HRMS analysis revealed that fraction C1 contained a complex mixture of multiple components, while fraction C2 showed a simplified profile dominated by a single major compound (Figure 1A; Supplementary Figure S3). Due to its higher potency and improved purity, fraction C2 was selected for further biological evaluation and chemical characterization. Fraction C2 also exhibited antiplasmodial activity against *P. falciparum* strains K1 and Dd2, with IC_50_ values of 24.50 µg/mL and 37.76 µg/mL, respectively (Table 1). No cytotoxicity was observed in HSF cells up to 200 µg/mL (CC_50_ > 200 µg/mL), resulting in selectivity indices (SI) greater than 30.53 for 3D7, 8.16 for K1, and 5.30 for Dd2. The resistant indices were calculated as 3.74 for K1 and 5.76 for Dd2. These results indicate that fraction C2 retained antiplasmodial activity with moderate potency and acceptable selectivity.

### 2.3. GNPS-Based Dereplication of Chemical Components in the Active Fraction C2

The active fraction C2 was analyzed using LC–HRMS to determine its chemical composition. The chromatographic profile (TIC) showed a dominant peak at a retention time of 9.77 min, indicating the presence of a major compound in the fraction (Figure 1A). MS/MS data obtained from fraction C2 were subjected to GNPS spectral library dereplication analysis. Among the returned annotations, a bile acid-related metabolite was suggested, with ursodeoxycholic acid (UDCA; C_24_H_40_O_4_; Figure 1B) returned as the closest spectral library match (Table S1). However, because the precursor ion detected in the experimental data differed from the library precursor ion of UDCA and no authentic standard comparison or spectroscopic confirmation was performed, the annotation was considered tentative. Therefore, the GNPS result was treated as a hypothesis-generating observation, and commercial UDCA was selected as a representative bile acid-related compound for subsequent biological evaluation.

### 2.4. In Vitro Antiplasmodial Activity of UDCA

Following GNPS-based annotation of a putative UDCA in fraction C2, a commercially available UDCA was evaluated to investigate whether a bile acid candidate with similar structural features could account for the observed antiplasmodial activity. UDCA showed antiplasmodial activity against all tested *P. falciparum* strains, with IC_50_ values of 4.68 ± 0.65 µg/mL (3D7), 12.89 ± 6.02 µg/mL (K1), and 21.13 ± 8.61 µg/mL (Dd2). These values are comparable to those of fraction C2 (6.55 ± 3.00, 24.50 ± 7.39, and 37.76 ± 4.17 µg/mL, respectively), indicating that UDCA may account, at least in part, for the activity observed in the fraction. Both UDCA and fraction C2 showed no cytotoxicity against HSF cells at concentrations up to 200 µg/mL (CC_50_ > 200 µg/mL). UDCA exhibited slightly higher selectivity indices (>42.74, >15.52, >9.47) compared to fraction C2 (>30.53, >8.16, >5.30), although both remained within a similar range. The resistant indices of UDCA (2.75 for K1 and 4.51 for Dd2) were comparable to those of fraction C2 (3.74 and 5.76, respectively). Reference compounds chloroquine (CQ) and artemisinin (ART) showed substantially lower IC_50_ values, consistent with their known potency. However, chloroquine displayed higher resistance indices, whereas UDCA maintained moderate resistance values. These results indicate that UDCA exhibits antiplasmodial activity at a level comparable to fraction C2 and likely contributes to its bioactivity.

**Figure 2:**
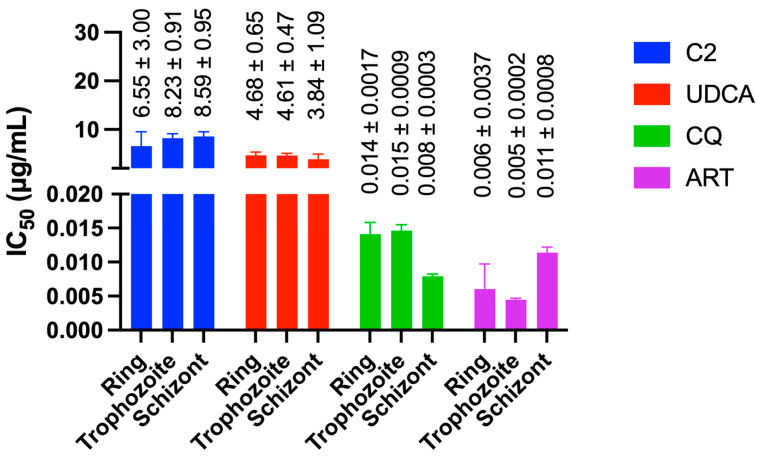
Stage-specific antiplasmodial activity of fraction C2 and ursodeoxycholic acid (UDCA) against *P. falciparum* 3D7 parasites. Synchronized parasites at ring, trophozoite, and schizont stages were exposed to fraction C2, UDCA, or reference control drugs for 72 h, followed by growth evaluation using the SYBR Green I assay. No statistically significant differences were observed among developmental stages for fraction C2, UDCA, or the reference drugs based on two-way ANOVA followed by Tukey’s multiple comparison test (*p* > 0.05). The numbers shown and graph data are presented as average IC_50_ ± SD from three independent experiments. Chloroquine (CQ); artemisinin (ART); half maximal inhibitory concentration (IC_50_).

To further evaluate stage-dependent susceptibility, synchronized *P. falciparum* 3D7 parasites were exposed to UDCA and fraction C2 during ring, trophozoite, and schizont stages (Figure 2). No statistically significant differences were observed among the tested developmental stages for either UDCA, fraction C2, or the reference control drugs based on two-way ANOVA followed by Tukey’s multiple comparison test (*p* > 0.05). These findings suggest that both UDCA and fraction C2 exhibit inhibitory activity across multiple intraerythrocytic developmental stages.

### 2.5. In Silico Drug Target Prediction and Docking Simulations of UDCA

**Figure 3:**
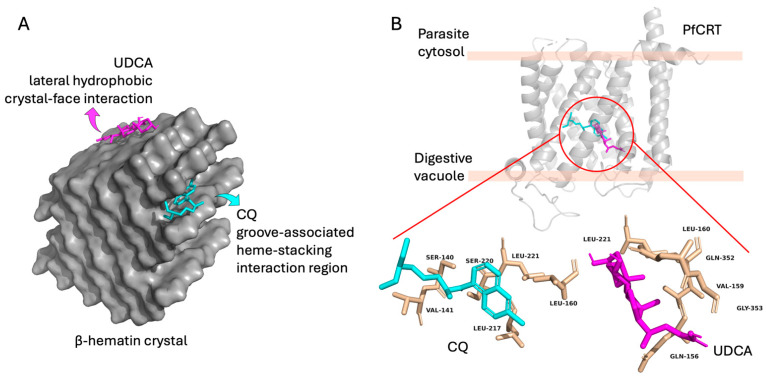
Comparative docking analysis of ursodeoxycholic acid (UDCA) and chloroquine (CQ) against β-hematin and PfCRT. (**A**) Docking visualization of UDCA (magenta) and CQ (cyan) on the β-hematin crystal surface. (**B**) Comparative docking analysis within the *P. falciparum* chloroquine-resistant transporter (PfCRT) cavity; the approximate membrane boundaries are indicated by horizontal bars.

To investigate whether UDCA may act through mechanisms distinct from classical quinoline antimalarial drugs, comparative docking analyses against β-hematin and the *P. falciparum* chloroquine resistance transporter (PfCRT) were performed using chloroquine (CQ) as a reference compound (Figure 3; Table 2). In the β-hematin model, both UDCA and CQ showed favorable predicted binding affinities and convolutional neural network (CNN) scores; however, their binding poses differed markedly. CQ localized within the canonical quinoline-associated groove region of the β-hematin crystal surface, whereas UDCA occupied a distinct surface-exposed interaction region (Figure 3A). These findings suggest that UDCA may interact with β-hematin through a binding mode different from the classical CQ-associated heme detoxification inhibition mechanism. Similarly, docking analysis against PfCRT demonstrated that UDCA localized within a cavity region partially overlapping with the CQ-binding region; however, the predicted molecular orientation and interaction profile differed from those of CQ (Figure 3B). Although both compounds interacted within the central PfCRT cavity and showed favorable docking scores (Table 2), distinct molecular poses and residue-contact patterns were observed between UDCA and CQ, suggesting that UDCA may not be strongly influenced by classical CQ resistance-associated transport mechanisms. Because UDCA retained antiplasmodial activity against the multidrug-resistant K1 and Dd2 strains, these findings support the possibility that UDCA may act through pathways partially distinct from conventional quinoline antimalarial drugs. Based on these observations, additional protein target prediction and docking analyses were subsequently performed to further investigate alternative mechanisms associated with UDCA antiplasmodial activity.

To identify potential molecular targets of UDCA, malaria-associated genes from GeneCards were compared with predicted targets for UDCA from SwissTargetPrediction. A total of 5263 genes were obtained from GeneCards and 106 predicted targets from SwissTargetPrediction, with 40 overlapping targets (0.7%). These 40 overlapping targets were subsequently analyzed using STRING to explore protein–protein interactions and to identify corresponding *P. falciparum* orthologs. This analysis resulted in 11 mapped *P. falciparum* proteins with varying levels of sequence homology and network connectivity (Table 3). Among these, three targets were selected for further study based on their relatively higher sequence homology and functional relevance: glucose-6-phosphate dehydrogenase/6-phosphogluconolactonase (PfGluPho; Q8IKU0, 39.65%), mitogen-activated protein kinase (PfMAPK; Q8ILF0, 39.49%), and protein farnesyltransferase subunit beta (PfPFT-β; Q8IHP6, 33.18%). These proteins are associated with key metabolic and signaling pathways and showed moderate node degrees within the interaction network. Other identified targets showed lower sequence homology or less relevance for parasite-specific processes and were therefore not prioritized. Based on this selection, PfGluPho, PfMAPK, and PfPFT-β were chosen as representative targets for subsequent molecular docking analysis with UDCA.

**Figure 4:**
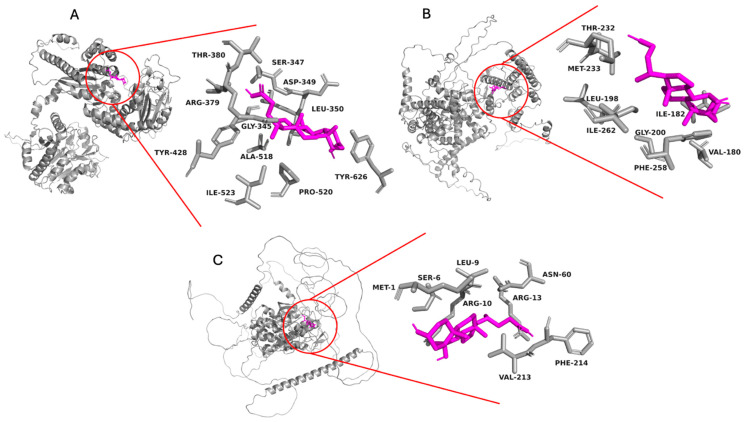
Binding modes and predicted receptor–ligand interactions within 4 Å of ursodeoxycholic acid (UDCA) for (**A**) *P. falciparum* glucose-6-phosphate dehydrogenase/6-phosphogluconolactonase (PfGluPho; AlphaFold ID: AF-Q8IKU0), (**B**) *P. falciparum* mitogen-activated protein kinase (PfMAPK; AlphaFold ID: AF-Q8ILF0), and (**C**) *P. falciparum* protein farnesyltransferase subunit beta (PfPFT-β; AlphaFold ID: AF-Q8IHP6). Receptor structures are shown in gray and UDCA is colored magenta.

To investigate the potential binding of UDCA to the selected targets, molecular docking was performed against PfGluPho, PfMAPK, and PfPFT-β. The docking results showed that UDCA exhibited favorable binding affinities across all three proteins (Table 4; Figure 4), with the strongest interaction observed for PfGluPho (−8.04 kcal/mol), followed by PfPFT-β (−6.53 kcal/mol) and PfMAPK (−5.46 kcal/mol). The corresponding convolutional neural network (CNN) scores were 0.9450, 0.8384, and 0.8251, respectively, indicating reliable docking poses. For PfGluPho, UDCA formed multiple hydrogen bond interactions with key residues, including Ser347, Asp349, Leu350, Arg379, and Thr380. In addition, hydrophobic interactions were observed with Leu350, Arg379, Ile523, and Tyr626, suggesting stable binding within the active region. The relatively strong binding affinity and high CNN score support PfGluPho as a potential target. In the case of PfMAPK, UDCA formed hydrogen bonds with Thr232 and Met233, along with hydrophobic interactions involving Val180, Ile182, Thr232, Phe258, and Ile262. Although the binding affinity was lower than that of PfGluPho, the interaction profile indicates a possible binding mode within the kinase domain. For PfPFT-β, UDCA formed through hydrogen bonds with Arg10 and Phe214, and hydrophobic contacts with Met1, Leu9, Arg10, Arg13, and Val213. The binding affinity (−6.53 kcal/mol) and interaction pattern suggest moderate binding stability. Comparison with reference inhibitors (provided in Supplementary Figure S4) showed that UDCA exhibited comparable binding affinity trends, although slightly lower than the known inhibitors for each respective target. Overall, these results indicate that UDCA can interact with multiple parasite proteins, with the strongest predicted binding toward PfGluPho, supporting its potential multi-target mode of action.

### 2.6. Effects of UDCA on P. yoelii 17XNL-Infected Mice

**Figure 5:**
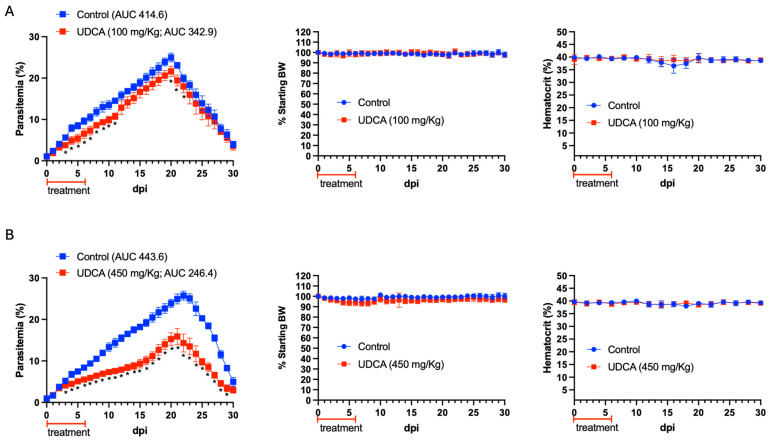
Effects of 7-day treatment with ursodeoxycholic acid (UDCA) on parasitemia levels in *P. yoelii* 17XNL-infected C57BL/6 mice at doses of (**A**) 100 mg/kg and (**B**) 450 mg/kg. Parasitemia progression, AUC, body weight (BW), and hematocrit are shown. Statistically significant differences relative to the untreated control are indicated by asterisks (* *p* < 0.05). Data are presented as mean ± SD and were analyzed using two-way ANOVA followed by Sidak’s multiple comparisons test. AUC, area under the curve.

The in vivo antiplasmodial effect of UDCA was evaluated in *P. yoelii* 17XNL-infected mice by monitoring parasitemia, area under the parasitemia–time curve (AUC), body weight, and hematocrit during and after the 7-day oral treatment period. At 100 mg/kg (Figure 5A), UDCA produced a moderate reduction in parasitemia compared with the untreated control. In the control group, parasitemia gradually increased and reached a peak of approximately 24–25% around 19–20 dpi, whereas the UDCA-treated group reached a lower peak of about 20–21%. The overall parasite burden was also reduced, as reflected by a lower AUC value in the treated group (342.9) compared with the control (414.6). The difference was most apparent during the ascending phase of infection and around the peak parasitemia period, where several time points showed statistical significance. After the peak, parasitemia declined in both groups and reached similarly low levels by the end of the observation period.

At 450 mg/kg (Figure 5B), the reduction in parasitemia was more evident. While the control group again showed a progressive increase in parasitemia with a peak of approximately 25–26%, the UDCA-treated group remained clearly lower throughout most of the infection course and peaked at around 15–16%. This effect was also supported by the AUC analysis, which showed a marked reduction from 443.6 in the control group to 246.4 in the UDCA-treated group. Significant differences were observed across multiple days, indicating a stronger suppressive effect at 450 mg/kg than at 100 mg/kg.

Changes in body weight were minimal in both treatment groups. At 100 mg/kg, body weight remained close to the starting value throughout the experiment and was comparable to the control group. A similar pattern was observed at 450 mg/kg, although a slight transient decrease was seen during the treatment period. Overall, no marked body weight loss was detected. Likewise, hematocrit values remained generally stable in both control and UDCA-treated mice. In the 100 mg/kg group, a temporary decrease was observed around the middle of infection, but the values recovered and remained comparable to those of the control group thereafter. In the 450 mg/kg group, hematocrit remained nearly unchanged throughout the study and did not differ significantly from the control.

## 3. Discussion

This study identified an antiplasmodial active fraction from *Streptomyces* sp. D10, for which GNPS dereplication returned UDCA as the closest library match among the detected metabolites. The activity of the fraction C2 and the comparable efficacy of the commercial UDCA standard suggest that UDCA and/or structurally related bile acid derivatives may contribute, at least in part, to the observed activity, while additional components or interactions within the fraction cannot be excluded. Although the active metabolite present in fraction C2 remains unconfirmed, the putative annotation is also consistent with previous reports showing that bile acid–like compounds can be produced by *Streptomyces* species [16]. Several studies have described microbial transformation [17] or de novo biosynthesis [16] of bile acid derivatives by actinomycetes, suggesting that such metabolites are not restricted to mammalian systems. In actinomycetes, structurally diverse secondary metabolites are often associated with environmental adaptation, chemical competition, and stress-response regulation [18,19]. Therefore, bile acid-like metabolites produced or transformed by *Streptomyces* species may contribute to ecological fitness and microbial survival.

UDCA is known for its anti-inflammatory, antioxidant, and cytoprotective properties and is clinically used for the treatment of biliary tract diseases [20,21]. Its biological effects are associated with multiple mechanisms, including modulation of cell signaling, maintenance of mitochondrial integrity, and regulation of cellular stress responses [22]. In *Plasmodium*, redox balance, mitochondrial function, and membrane integrity are critical for parasite survival, as the parasite is continuously exposed to oxidative stress during intraerythrocytic development and relies on tightly regulated antioxidant systems for viability [23]. Disruption of these processes, including NADPH-dependent redox pathways and mitochondrial metabolism, can impair parasite growth and represents a validated strategy for antimalarial intervention [24]. Therefore, bile acid–like molecules such as UDCA may represent a relevant chemical class for further investigation in antiparasitic drug discovery.

In vitro, UDCA showed moderate activity against *P. falciparum* 3D7 and retained activity against multidrug-resistant strains K1 and Dd2, with relatively low resistance indices compared to CQ. In addition, stage-specific susceptibility assays demonstrated that both fraction C2 and UDCA inhibited parasite growth across ring, trophozoite, and schizont stages without significant stage-dependent differences, indicating activity throughout multiple intraerythrocytic developmental stages. While the potency of UDCA was lower than that of standard antimalarials such as CQ and ART, its selectivity profile was favorable, with no cytotoxicity observed up to 200 µg/mL. This suggests that UDCA may represent a chemically distinct scaffold with a different mechanism of action, rather than a direct competitor to existing high-potency drugs.

Comparative docking analyses against β-hematin and PfCRT using CQ as a reference compound suggested that UDCA may interact with parasite targets through mechanisms partially distinct from classical quinoline antimalarial drugs. Although UDCA showed favorable predicted interactions with both β-hematin and PfCRT, its binding poses differed from those observed for CQ. In the β-hematin model, UDCA occupied a distinct surface-exposed crystal interaction region rather than the canonical CQ-associated groove region. This mode of interaction differs from established models in which quinoline antimalarials inhibit β-hematin growth by adsorbing to specific crystal faces or growth steps, thereby blocking further crystal elongation [25]. Similarly, within the PfCRT cavity, UDCA adopted a different molecular orientation and interaction profile compared with CQ. These observations suggest that the retained activity of UDCA against the multidrug-resistant K1 and Dd2 strains may not primarily depend on classical chloroquine-associated resistance pathways, supporting the possibility that UDCA may exert antiplasmodial effects through alternative molecular targets and biological pathways.

Target prediction and docking analysis suggest that UDCA may interact with multiple parasite proteins, including PfGluPho, PfMAPK, and PfPFT-β. Among these, PfGluPho showed the strongest predicted binding affinity and highest CNN score, supported by multiple hydrogen bonds and hydrophobic interactions. PfGluPho is a bifunctional enzyme involved in the pentose phosphate pathway, which plays a central role in maintaining intracellular redox balance through NADPH production [26]. In *Plasmodium*, this pathway is essential for protecting the parasite from oxidative stress generated during hemoglobin digestion and host immune responses. Inhibition of PfGluPho can disrupt NADPH supply, leading to accumulation of reactive oxygen species and impaired parasite survival [26]. Therefore, the interaction of UDCA with PfGluPho provides a plausible explanation for its antiplasmodial activity, particularly under conditions in which oxidative stress is critical for parasite viability.

PfMAPK (mitogen-activated protein kinase) is associated with signaling pathways that regulate parasite development, differentiation, and cell cycle progression. Although the MAPK pathways in *Plasmodium* are less complex than in higher eukaryotes, they remain important for coordinating responses to environmental changes during the intraerythrocytic cycle. Inhibition of PfMAPK may interfere with parasite growth and stage progression, potentially leading to delayed development or reduced replication [27]. The moderate binding affinity of UDCA to PfMAPK suggests that it may contribute to parasite growth suppression through partial disruption of signaling pathways.

PfPFT-β is involved in post-translational modification of proteins through farnesylation, a process required for the proper localization and function of several essential proteins, including small GTPases [28]. In *Plasmodium*, farnesylation is critical for membrane trafficking, signal transduction, and parasite survival. Inhibition of PfPFT-β can disrupt these processes, leading to impaired protein targeting and reduced parasite viability [29]. The interaction of UDCA with PfPFT-β, although moderate in binding affinity, suggests a possible contribution to its multi-target activity. Taken together, these findings indicate that UDCA may exert its antiplasmodial effects through a multi-target mechanism involving disruption of redox homeostasis (PfGluPho), signaling pathways (PfMAPK), and protein modification processes (PfPFT-β). This multi-target profile may partly explain its activity against drug-resistant strains and could be advantageous in reducing the likelihood of resistance development. However, the potential targeting of G6PD-related pathways requires careful consideration. In humans, glucose-6-phosphate dehydrogenase (G6PD) deficiency is a common genetic condition that affects redox homeostasis in erythrocytes. Drugs that interfere with this pathway can induce hemolysis in G6PD-deficient individuals [30]. Although UDCA is an FDA-approved drug with a well-established safety profile for liver-related disorders [31], its potential interaction with parasite G6PD-like enzymes raises the need to evaluate its effects in the context of human G6PD deficiency. The low hemolysis rate in vitro and stable hematocrit levels in vivo are encouraging, but more specific studies are required to confirm safety in this population.

The in vivo results using the *P. yoelii* 17XNL model further support the biological relevance of UDCA. A dose-dependent reduction in parasitemia was observed, particularly at 450 mg/kg, where both peak parasitemia and overall parasite burden (AUC) were clearly reduced. Importantly, this effect was achieved without significant changes in body weight or hematocrit, suggesting that UDCA was well tolerated under the tested conditions. However, parasite clearance was not achieved, indicating that UDCA alone may not be sufficient as a standalone therapy but could be considered as part of a combination strategy. Because the present study used the non-lethal *P. yoelii* 17XNL in C57BL/6J mice model, which allows extended monitoring of parasitemia progression and treatment responses, additional studies using more virulent rodent malaria strains, such as *P. yoelii* 17XL, or other experimental malaria models will be valuable to further evaluate the strain dependency and broader in vivo antimalarial potential of UDCA.

Another important aspect of this study is that GNPS dereplication of the active fraction suggested a bile acid-related metabolite, which prompted investigation of bile acid-like compounds as potential antiplasmodial agents. The biological activity observed for commercial UDCA supports the possibility that bile acid-related metabolites may contribute to the antiplasmodial activity associated with the active fraction. This also highlights the possibility that microbial-derived metabolites with known pharmacological properties may have additional, previously unrecognized biological activities. Previous studies have demonstrated that bile acid-related compounds can be generated through microbial biotransformation or biosynthetic processes in actinomycetes and other microorganisms [16,17]. Therefore, optimization of fermentation conditions, exploration of alternative microbial hosts, or metabolic engineering approaches may improve UDCA production yield and potentially enable the generation of structurally related bile acid derivatives with enhanced antiparasitic properties. Overall, the results indicate that UDCA exhibits moderate antiplasmodial activity in vitro and in vivo, with a favorable safety profile under the tested conditions. Its activity against resistant strains and predicted multi-target interactions suggest potential as a lead compound or as part of combination therapy. Further studies are needed to clarify its mechanism of action, evaluate its activity in combination with existing antimalarial drugs, definitively identify the active metabolite(s) present in fraction C2, and assess its safety in the context of G6PD deficiency.

## 4. Materials and Methods

### 4.1. Sampling and Isolation of Actinomycetes

Soil samples were collected from Tujiin Nars National Park, Altanbulag soum, Selenge Province, Mongolia (GPS: 50.192862° N, 106.444618° E), following removal of surface debris and sampling at a depth of 5–10 cm. Selenge Province was selected because it represents one of the major forested regions of Mongolia and is considered a potential source of diverse microbial communities and bioactive actinomycetes [6]. The samples were subsequently air-dried, and serial dilutions ranging from 10^−1^ to 10^−8^ were prepared using sterile 0.9% NaCl solution (Wako, Osaka, Japan). A volume of 0.1 mL from suitable dilutions was spread onto Gauze No.1 medium (comprising 20 g starch, 1 g/L KNO_3_, 0.5 g/L NaCl, 0.5 g/L K_2_HPO_4_, 0.5 g/L MgSO_4_, 0.01 g/L FeSO_4_, and 10 g/L agar; Wako, Osaka, Japan) and YM agar (containing 3 g/L yeast extract, 3 g/L malt extract, 5 g/L peptone, 10 g/L glucose, and 15–20 g/L agar; Wako, Osaka, Japan), both supplemented with cycloheximide (50 mg/L; Wako, Osaka, Japan) to suppress fungal contamination. Plates were incubated at 28 °C for 7–14 days to allow colony development and facilitate isolation of pure cultures. Colonies exhibiting actinomycete-like morphology, including dry and powdery colony surfaces with filamentous growth characteristics, were selected and purified through repeated streaking on agar media. Genomic DNA of four independent actinomycete isolates was extracted from the purified actinomycete isolates using a commercial kit (Guangzhou Dongsheng Biotech Co., Ltd., Guangzhou, China). DNA quality and concentration were evaluated using a NanoDrop 1000 spectrophotometer (Thermo Fisher Scientific, Wilmington, DE, USA). The extracted genomic DNA showed concentrations ranging from 20.9 to 29.5 ng/μL, with purity ratios within acceptable ranges for downstream PCR amplification and sequencing. The 16S rRNA gene was amplified by PCR using primers 8F (Forward; 5′-AGA GTT TGA TCC TGG CTC AG-3′) and 1492R (Reverse; 5′-TAC GGC TAC CTT GTT ACG ACT T-3′) [32]. The resulting PCR products were purified with a Qiagen gel extraction kit (Qiagen, Hilden, Germany) and sequenced using an ABI 3130xl Genetic Analyzer (Applied Biosystems, Foster City, CA, USA). Sequencing analysis was successfully obtained for all isolates. The resulting sequences consistently indicated affiliation with the genus *Streptomyces* (Supplementary Figure S1). For strain D10, repeated sequencing was performed to improve sequence quality, and the final sequence data used for analysis were obtained after at least three sequencing attempts. Sequence data were analyzed using BioEdit version 7.2 and MEGA version 12 software.

### 4.2. Fermentation and Extraction

The D10 strain was cultured in ISP 2 (International Streptomyces Project-2; BD Difco, Sparks, MD, USA) broth at 28 °C for 7 days to produce crude metabolites. Fermentation was performed in 500 mL Erlenmeyer flasks containing 250 mL of ISP 2 medium, incubated at 28 °C with agitation at 200 rpm for 7 days. After cultivation, the culture broth was centrifuged (2236× *g*, 10 min) to separate the supernatant from the mycelial biomass. The supernatant was then extracted with an equal volume of ethyl acetate, and the organic phase was concentrated using a Ren-1000 rotary evaporator at 50 °C (IWAKI, Shizuoka, Japan). Crude extract was subsequently obtained (2 g) and used for antiprotozoal evaluation.

### 4.3. Fractionation and Compound Identification

Compound separation was performed through a two-step chromatographic approach involving column chromatography using silica gel 60 (Kanto Chemical Co., Inc., Tokyo, Japan), followed by thin-layer chromatography (TLC) on precoated silica gel 60 F254 plates (Merck, Darmstadt, Germany). The crude extract was chromatographed on a silica gel column using 10% stepwise of n-hexane-EtOAc (100:0–0:100, each 100 mL), then a mixture of EtOAc-MeOH (50:50, 100 mL), and finally MeOH (100 mL) to give 13 fractions (Fr. 1–1 to 1–13). Fractions 1–5 to 1–7 were combined and further chromatographed on a silica gel column using 10% stepwise of CHCl_3_-EtOAc (100:0–0:100, each 50 mL) to afford 11 fractions (Fr. 2–1 to 2–11). Fractions 2–4 and 2–5 were combined and were further subjected to preparative thin-layer chromatography with a chloroform–methanol mixture (9:1, *v*/*v*) as the mobile phase for fractionation. After TLC separation (Supplementary Figure S2), the desired bands were scraped from the plates and extracted with ethyl acetate. The silica residues were subsequently removed by centrifugation. The resulting purified fractions C1 (90 mg) and C2 (50 mg) were then analyzed by liquid chromatography–high resolution mass spectrometry (LC-HRMS) for purity check and compound identification (Supplementary Figure S3).

### 4.4. LC-HRMS Analysis

The chemical composition of fractions C1 and C2 was characterized using ultra-performance liquid chromatography (UPLC) coupled with a Synapt G2 HDMS mass spectrometer (Waters, Milford, MA, USA). Mass spectrometric detection was carried out in positive electrospray ionization (ESI) mode. The operating parameters included a capillary voltage of 2.0 kV, cone voltage of 30 V, desolvation temperature of 550 °C, and source temperature of 120 °C. The desolvation gas flow was maintained at 90 L/h, and ions were monitored within an *m*/*z* range of 150–800. Leucine enkephalin was used as an internal standard for mass calibration in the Waters LC–HRMS system. Chromatographic separation was performed on a Mightysil RP-18 GP II column (50 × 2.0 mm; Kanto Chemical, Tokyo, Japan). A gradient elution system was applied using water containing 0.1% formic acid (solvent A) and acetonitrile containing 0.1% formic acid (solvent B). The gradient program was set as follows: 0–1 min, 1% B; 1–6 min, increased linearly to 90% B; 7–12 min, further increased to 99% B; 12–16 min, held at 99% B; 16–17 min, decreased to 1% B; and 17–20 min, maintained at 1% B. The acquired MS data for C2 was subsequently analyzed using GNPS-based metabolomic tools to annotate and identify the major constituents.

### 4.5. Chemicals

Standard ursodeoxycholic acid (UDCA) was purchased from Adipogen Life Sciences (San Diego, CA, USA). Chloroquine diphosphate (CQ) and artemisinin (ART) were obtained from Sigma-Aldrich (St. Louis, MO, USA) and used as reference compounds. Dimethyl sulfoxide (DMSO; Wako, Osaka, Japan) was used as the vehicle control.

### 4.6. In Vitro Antiplasmodial Activity

Cultures of *P. falciparum* 3D7 (CQ-sensitive), K1 (CQ- and pyrimethamine resistant), and Dd2 (multidrug-resistant; including CQ, mefloquine, and pyrimethamine) strains were maintained in human erythrocytes at 2% hematocrit using RPMI 1640 medium (Sigma-Aldrich). Parasites were synchronized to the ring stage using sorbitol treatment, achieving a synchronization level above 90%. For stage-specific susceptibility assays, sorbitol-synchronized *P. falciparum* 3D7 parasites were exposed to UDCA during ring (0–2 h), trophozoite (8–10 h), or schizont (24–26 h) stages. Crude extracts, fractions, or compounds at the required concentrations were dispensed into 96-well plates (50 μL per well), followed by the addition of infected erythrocytes (50 μL per well; parasitemia 0.5%, hematocrit 2%). Crude extracts, fractions, and UDCA were tested at concentrations ranging from 100 to 0.78 μg/mL using eight serial two-fold dilutions, while the positive control drug was tested at concentrations ranging from 0.1 to 0.00078 μg/mL. The selected concentration ranges were designed to ensure appropriate coverage for accurate IC_50_ determination and to obtain reliable sigmoidal dose–response curves across samples with varying antiplasmodial potencies. Plates were incubated at 37 °C for 72 h.

Parasite proliferation was assessed by adding 100 μL of lysis buffer containing SYBR Green I nucleic acid stain (10,000×; Lonza Rockland, Rockland, ME, USA). Fluorescence intensity was measured using a SpectraMax iD5 (Molecular Devices, San Jose, CA, USA) with excitation and emission wavelengths of 485 nm and 518 nm, respectively. The percentage of growth inhibition was calculated by comparing fluorescence signals between treated and untreated control wells. Background signals from uninfected erythrocytes and compound-related fluorescence were subtracted prior to analysis.

### 4.7. In Vitro Cytotoxicity and Hemolysis Assays

In this study, human skin fibroblast cells (HSF; NB1RGB, RCB0222, RIKEN BRC, Ibaraki, Japan) were cultured in DMEM supplemented with 10% fetal bovine serum (FBS) and 1% penicillin–streptomycin (Wako). Cell suspensions (1 × 10^5^ cells/mL) were seeded into 96-well plates and incubated at 37 °C in a 5% CO_2_ incubator for 48 h. The tested samples, including compounds, fractions, and crude extracts, were dissolved in DMSO and applied at a maximum final concentration of 200 μg/mL and serially 2× diluted to final concentrations ranging from 200 to 1.56 μg/mL (eight concentrations). The selected concentration range was designed to provide sufficient coverage for accurate CC_50_ determination and reliable sigmoidal dose–response curve fitting across samples, while maintaining consistency with the concentration ranges used in the antiplasmodial assays. After 72 h of treatment, cell viability was evaluated by adding Cell Counting Kit-8 (CCK-8; Dojindo, Kumamoto, Japan), and absorbance was measured at 450 nm using a SpectraMax iD5. Hemolysis assay against human red blood cells was evaluated at 100 μg/mL for each fraction or compound as described previously [33].

### 4.8. In Silico Target Prediction and Molecular Docking

Malaria-associated genes were obtained from GeneCards, where these genes are defined as those functionally linked to *Plasmodium* biology, parasite survival, or antimalarial relevance based on curated annotations and literature. The UDCA predicted targets were identified using SwissTargetPrediction [34]. These targets were then matched with *P. falciparum* orthologs using UniProt and PlasmoDB to ensure parasite-specific identification. The overlapping targets were further analyzed using STRING (Search Tool for the Retrieval of Interacting Genes/Proteins; species: *Homo sapiens*, confidence ≥ 0.4) to infer corresponding *P. falciparum* homologs [35]. The top three predicted targets, based on their percent homology, were selected for molecular docking studies.

For molecular docking, conformers of UDCA were generated using RDKit with the ETKDGv3 algorithm (Experimental Torsion Knowledge Distance Geometry, version 3) [36] and subsequently optimized using the MMFF (Merck Molecular Force Field). Conformational clustering was performed using DBSCAN (Density-Based Spatial Clustering of Applications with Noise) [37,38], resulting in 14 clusters from which representative structures were selected for docking. Each conformer was docked using Gnina [39,40,41,42], which combines traditional scoring functions with a convolutional neural network (CNN)-based scoring system. The CNN score (ranging from 0 to 1) reflects the predicted likelihood of a correct binding pose, with higher values indicating greater confidence in the docking result. Comparative docking analyses against β-hematin and the *P. falciparum* chloroquine resistance transporter (PfCRT) were performed to evaluate whether UDCA may interact through mechanisms distinct from classical quinoline antimalarial drugs. The β-hematin crystal structure was obtained from the Cambridge Crystallographic Data Centre (CCDC 162267) [43] and expanded into a 2 × 2 × 2 hemozoin supercell using VESTA version 3 software [44]. The PfCRT structure was retrieved from the RCSB Protein Data Bank (PDB ID: 6UKJ), corresponding to the chloroquine-resistant 7G8 isoform [45]. Docking grids were defined to encompass the entire receptor structure. Four predicted UDCA protein target structures were retrieved from the AlphaFold database, including *P. falciparum* glucose-6-phosphate dehydrogenase/6-phosphogluconolactonase (PfGluPho; AF-Q8IKU0), mitogen-activated protein kinase (PfMAPK; AF-Q8ILF0), and protein farnesyltransferase subunit beta (PfPFT-β; AF-Q8IHP6). The docking centers were defined as follows: PfGluPho (x, y, z: 7.555, −16.756, −12.839), PfMAPK (x, y, z: 4.335, 5.077, 20.264), and PfPFT-β (x, y, z: −24.747, 30.206, −3.371), with a box size of 20 Å in each dimension. Protein–ligand interactions were analyzed using PLIP (Protein–Ligand Interaction Profiler) [46]. Known inhibitors, SBI-0797750 (PfGluPho) [47], SB203580 (PfMAPK) [48], and BMS-386914 (PfPFT-β) [49], were included in the simulations as references. All molecular visualizations were generated using PyMOL version 3.1.6.1.

### 4.9. Mice and In Vivo Infection

In vivo experiments were conducted using twelve-week-old male C57BL/6J mice (average body weight ~30 g; Japan CLEA, Tokyo, Japan). Animals were housed in groups of six per cage under controlled environmental conditions (24 °C, 50% relative humidity, 12 h light–dark cycle) with ad libitum access to water and a standard rodent diet (CLEA Rodent Diet CE-2). All procedures were approved by the institutional animal ethics committee and performed in accordance with relevant guidelines.

The non-lethal rodent malaria parasite *P. yoelii* 17XNL was revived from cryopreserved infected erythrocytes by passage in mice. For infection, each mouse received an intraperitoneal injection of 1 × 10^7^ infected erythrocytes under isoflurane anesthesia, defined as day 0 post-infection (dpi). The experimental unit was an individual mouse. Mice were randomly assigned to control and treatment groups (*n* = 6 per group; total *n* = 12 per experiment), and the study was conducted in two independent experiments. Randomization was performed without a specific sequence generation method. No blinding was applied during allocation, conduct, or outcome assessment.

Parasitemia was assessed using Giemsa-stained thin blood smears prepared from 2 μL of tail blood. Treatment was initiated when parasitemia reached approximately 1%. Mice received oral administration of vehicle (1× PBS) or UDCA at doses of 100 or 450 mg/kg once daily for 7 consecutive days (0–6 dpi). The selected UDCA dose range was based on previously published reports demonstrating that oral administration of UDCA at doses up to 450 mg/kg/day in mice was generally well tolerated without severe toxicity [50]. Parasitemia and survival were monitored daily up to 30 dpi, and hematocrit levels were measured every other day.

No predefined inclusion or exclusion criteria were applied, and all animals were included in the analysis. No animals were excluded during the experiment. The sample size was determined based on commonly used group sizes in rodent malaria studies and ethical considerations to minimize animal use; no a priori power calculation was performed.

### 4.10. Statistical Analysis

Half-maximal inhibitory (IC_50_) and cytotoxic (CC_50_) values were determined from three independent experiments by applying nonlinear regression analysis, plotting the logarithm of compound concentration against the percentage inhibition of parasite growth or cell viability. Calculations were performed using GraphPad Prism 10 (GraphPad Software, Inc., La Jolla, CA, USA). Statistical comparisons between groups were carried out using two-way ANOVA, followed by Tukey’s or Sidak’s multiple comparison test. A *p*-value < 0.05 was considered statistically significant and is indicated by an asterisk along with the corresponding statistical test.

## 5. Conclusions

GNPS dereplication of an antiplasmodial active fraction from *Streptomyces* sp. D10 returned UDCA as the closest spectral library match, providing a hypothesis-generating basis for biological evaluation. Commercial UDCA exhibited moderate antiplasmodial activity in vitro and in vivo with low cytotoxicity and retained activity against multidrug-resistant *P. falciparum* strains. In silico analyses further suggested a potential multi-target mode of action involving PfGluPho, PfMAPK, and PfPFT-β, with interaction profiles distinct from chloroquine in β-hematin and PfCRT models. While the active metabolite present in fraction C2 remains unconfirmed, the results suggest that bile acid-related compounds may contribute to the observed bioactivity and merit further investigation as potential antimalarial scaffolds.

## Figures and Tables

**Table 1:** In vitro activities of the fraction and compounds tested in this study against *P. falciparum*, HSF cells, and RBC.

Sample	IC_50_ *P. falciparum* (μg/mL) [Hill Slope]			CC_50_ HSF Cells (μg/mL)	RI		SI			RBC Hemolysis Rate at 100 μg/mL (%)
	3D7	K1	Dd2		K1	Dd2	3D7	K1	Dd2	
C2	6.55 ± 3.00 [1.69 ± 0.34]	24.50 ± 7.39 [1.21 ± 0.05]	37.76 ± 4.17 [1.09 ± 0.61]	>200	3.74	5.76	>30.53	>8.16	>5.30	1.642 ± 0.362
UDCA	4.68 ± 0.65 [2.89 ± 0.20]	12.89 ± 6.02 [1.32 ± 0.13]	21.13 ± 8.61 [0.92 ± 0.24]	>200	2.75	4.51	>42.74	>15.52	>9.47	5.022 ± 0.527
Chloroquine	0.014 ± 0.002 [3.64 ± 0.79]	0.524 ± 0.300 [3.78 ± 1.16]	0.461 ± 0.020 [1.04 ± 0.06]	10.68 ± 3.51	37.43	32.93	762.86	20.38	23.17	1.262 ± 0.553
Artemisinin	0.006 ± 0.004 [2.10 ± 1.11]	0.007 ± 0.004 [1.79 ± 0.27]	0.013 ± 0.002 [1.57 ± 0.05]	43.20 ± 8.68	1.17	2.17	7200.00	6171.43	3323.08	0.586 ± 0.369

Values are presented as the average ± SD of at least three independent experiments. IC_50_, half-maximal inhibitory concentration. UDCA, ursodeoxycholic acid; CC_50_, half-maximal cytotoxic concentration; HSF, human skin fibroblast; RBC, red blood cell; RI, resistant index (ratio between IC_50_ of 3D7 with IC_50_ of K1 or Dd2); SI, selectivity index (ratio between IC_50_ and CC_50_).

**Table 2:** Interactions of chloroquine (CQ) and ursodeoxycholic acid (UDCA) from comparative docking simulations.

Receptor	Ligand	
	CQ	UDCA
β-hematin		
Binding affinity (kcal/mol)	−7.50	−10.00
CNN score	0.9451	0.8951
PfCRT (6UKJ)		
Binding affinity (kcal/mol)	−5.93	−7.01
CNN score	0.8053	0.9231
Hydrogen bond interactions	Ser140, Ser220, Gln253	Gln352, Gly353
Hydrophobic interactions	Val141, Leu160, Leu217, Leu221	Gln156, Val 159, Leu160, Leu221

**Table 3:** Predicted potential target genes of ursodeoxycholic acid (UDCA) from protein–protein interaction (PPI) network analysis.

UniProt Code	Human Gene	UniProt Code	*P. falciparum* 3D7 Gene	% Homology	Node Degree in PPI
P11413	G6PD	Q8IKU0	PF14_0511	39.65	1
Q16539	MAPK14	Q8ILF0	PF3D7_1431500	39.49	3
P49356	FNTB	Q8IHP6	PF3D7_1147500	33.18	2
Q9UBT2	UBA2	Q8I553	PF3D7_1237000	32.24	1
Q9UBE0	SAE1	Q8IHS2	PF3D7_1144500	27.21	1
O75907	DGAT1	O97295	PF3D7_0322300	26.62	2
P12931	SRC	Q8IEG4	PF3D7_1315100	25.00	18
P35610	SOAT1	O97295	PF3D7_0322300	23.35	1
P61964	WDR5	C0H4D3	PF3D7_0510800	22.77	2
P49354	FNTA	Q8I503	PF3D7_1242600	22.19	2
P00533	EGFR	O96197	PF3D7_0211700	19.63	14

**Table 4:** Interactions of ursodeoxycholic acid (UDCA) from docking simulations.

Parameter	Receptor		
	PfGluPho PF14_0511 (AF-Q8IKU0)	PfMAPK PF3D7_1431500 (AF-Q8ILF0)	PfPFT-β PF3D7_1147500 (AF-Q8IHP6)
Binding affinity	−8.04 kcal/mol	−5.46 kcal/mol	−6.53 kcal/mol
CNN score	0.9450	0.8251	0.8384
Hydrogen bond interactions	Ser347, Asp 349, Leu350, Arg379, Thr380,	Thr232, Met233	Arg10, Phe 214
Hydrophobic interactions	Leu350, Arg379, Ile523, Tyr626	Val180, Ile182, Thr232, Phe258, Ile262	Met1, Leu9, Arg10, Arg13, Val213

## Data Availability

The original contributions presented in this study are included in the article. Further inquiries can be directed to the corresponding author.

## Supplementary Materials

The following supporting information can be downloaded at https://www.mdpi.com/article/10.3390/ph19060958/s1: Figure S1. Taxonomic characterization of strain D10 based on 16S rRNA gene analysis; Figure S2. Bioassay-guided fractionation of the crude extract from *Streptomyces* sp. D10; Figure S3. LC–HRMS total ion chromatograms (TIC) of fractions C1 and C2; Figure S4. Binding modes and predicted receptor–ligand interaction of UDCA and respective reference control for each receptor; Table S1: GNPS-based spectral annotation of major compounds detected in fraction C2.

## Abbreviations

The following abbreviations are used in this manuscript:

ART	Artemisinin
CC_50_	50% cytotoxic concentration
CNN	Convolutional neural network
CQ	Chloroquine
DBSCAN	density-based spatial clustering of applications with noise
DMSO	Dimethyl sulfoxide
dpi	Day post-infection
ETKDG	Experimental torsion knowledge distance geometry
FBS	Fetal bovine serum
FDA	Food and Drug Administration
G6PD	Glucose-6-phosphate dehydrogenase deficiency
GNPS	Global Natural Products Social Molecular Networking
HSF	Human skin fibroblast cells
IC_50_	50% inhibitory concentration
MMFF	Merck molecular force field
MW	Molecular weight
PfGluPho	*Plasmodium falciparum* glucose-6-phosphate dehydrogenase/6-phosphogluconolactonase
PfMAPK	*Plasmodium falciparum* mitogen-activated protein kinase
PfPFT-β	*Plasmodium falciparum* protein farnesyltransferase subunit beta
PLIP	Protein–ligand interaction profiler
RI	Resistance index
SI	Selectivity index
STRING	Search tool for the retrieval of interacting genes/proteins
UDCA	Ursodeoxycholic acid
