# Supplementary material for: In Vitro, In Silico, and In Vivo Evaluation of Antiplasmodial Activity of Ursodeoxycholic Acid Following GNPS Dereplication of an Active Streptomyces sp. Fraction
Source: Pharmaceuticals (Basel). 2026 Jun 20;19(6):958. doi: 10.3390/ph19060958 (PMC13304703; doi:10.3390/ph19060958)
Supplement: Supplementary file 1 [file pharmaceuticals-19-00958-s001.zip › pharmaceuticals-4322586-supplementary.pdf]

Supplementary Materials

In Vitro, *In Silico*, and *In Vivo* Evaluation of Antiplasmodial Activity of Ursodeoxycholic Acid Identified from *Streptomyces* sp.

Nanang R. Ariefta [1], Baldorj Pagmadulam [1,2], Takako Aboshi [3], and Yoshifumi Nishikawa [1,*]

[1] National Research Center for Protozoan Diseases, Obihiro University of Agriculture and Veterinary Medi-cine, Inada-cho, Obihiro 080-8555, Japan

[2] Laboratory of Microbial Synthesis, Institute of Biology, Mongolian Academy of Sciences, Bayanzurkh District, 12 Khoroo, Ulaanbaatar 13270, Mongolia

[3] Department of Life, Food, and Environmental Sciences, Faculty of Agriculture, Yamagata University, Wakaba-machi 1-23, Tsuruoka, Yamagata, 997-8555, Japan

*Correspondence: nisikawa@obihiro.ac.jp (Y.N.)

Table S1. GNPS-based spectral annotation of major compounds detected in fraction C2. Compounds were annotated based on GNPS library matching, and all hits are shown.

Spectrum ID	Compound Name	Adduct	Precursor *m/z*	Exact Mass	MQ Score	Library	Molecular Formula	InChIKey	Class	Subclass
CCMSLIB00000206218	Leucine Enkephalin*	[M+H]+	556.277	556.28	0.955	MassBank	$C_{28}H_{37}N_5O_7$	URLZCHNOLZSCCA	Peptides	Tripeptides
CCMSLIB00000084832	Ursodeoxycholic acid	[M+H]+	393.30	392.29	0.775	GNPS-NIH	$C_{24}H_{40}O_4$	RUDATBOHQWOJDD	Steroids	Cholane steroids
CCMSLIB00000084832	Ursodeoxycholic acid	[M+H]+	393.30	392.29	0.768	GNPS-NIH	$C_{24}H_{40}O_4$	RUDATBOHQWOJDD	Steroids	Cholane steroids

*Leucine enkephalin was used as an internal standard for mass calibration in the Waters LC–MS system and was not considered a sample-derived metabolite.

This identification data is available on GNPS website: https://gnps.ucsd.edu/ProteoSAFe/status.jsp?task=b9abadf83b574e26875441cdf8d8502a

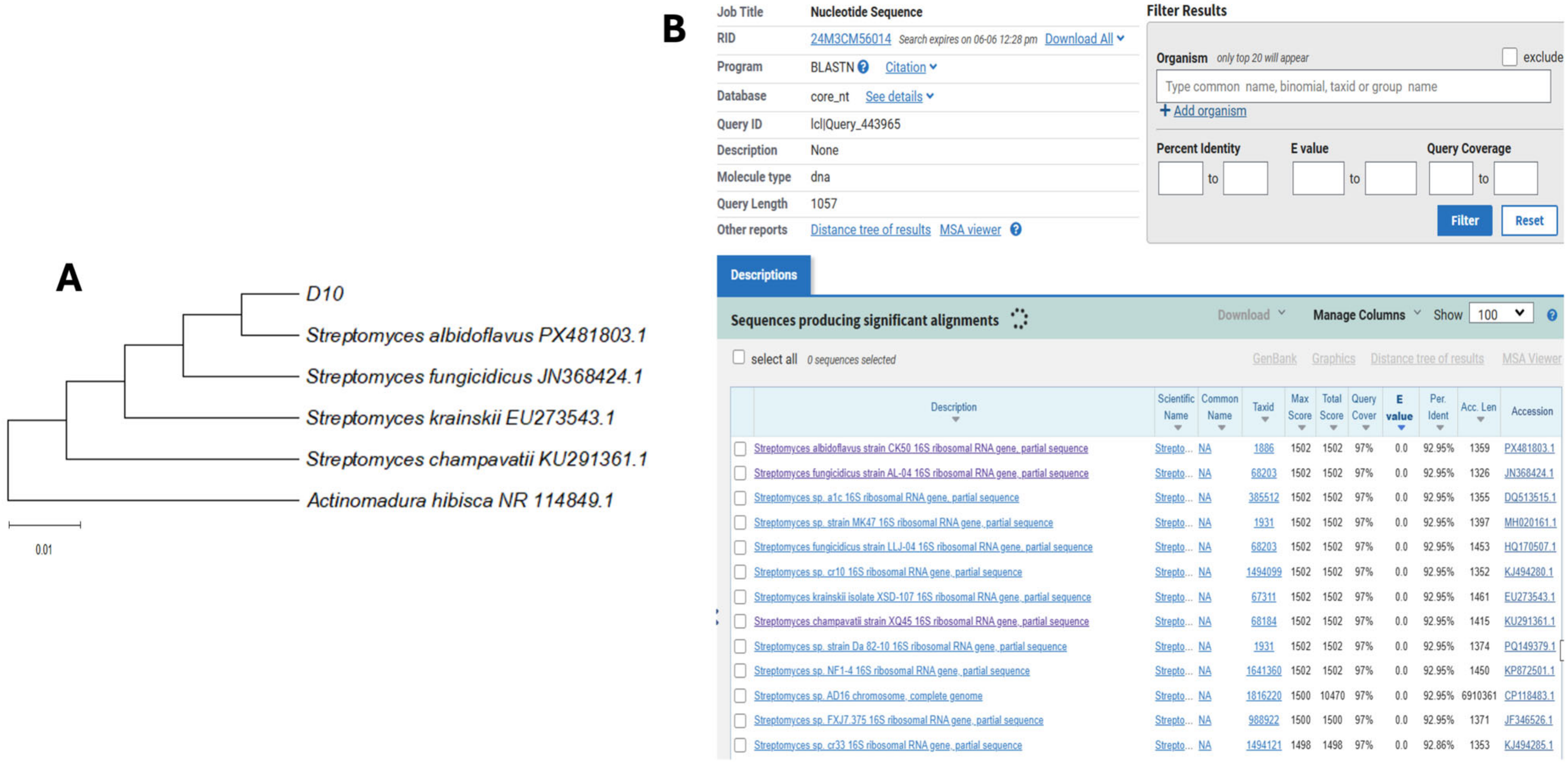

Figure S1. Taxonomic characterization of strain D10 based on 16S rRNA gene analysis. (A) Rooted neighbor-joining phylogenetic tree constructed from 16S rRNA gene sequences showing the relationship between strain D10 and representative *Streptomyces* species. The tree was generated using MEGA 12 with 1,000 bootstrap replications. (B) NCBI BLASTn similarity search results for the 16S rRNA gene sequence of strain D10. The closest match was *Streptomyces albidoflavus* (accession no. PX481803.1) with 92.95% sequence identity, supporting assignment to the genus *Streptomyces* but not species-level identification.

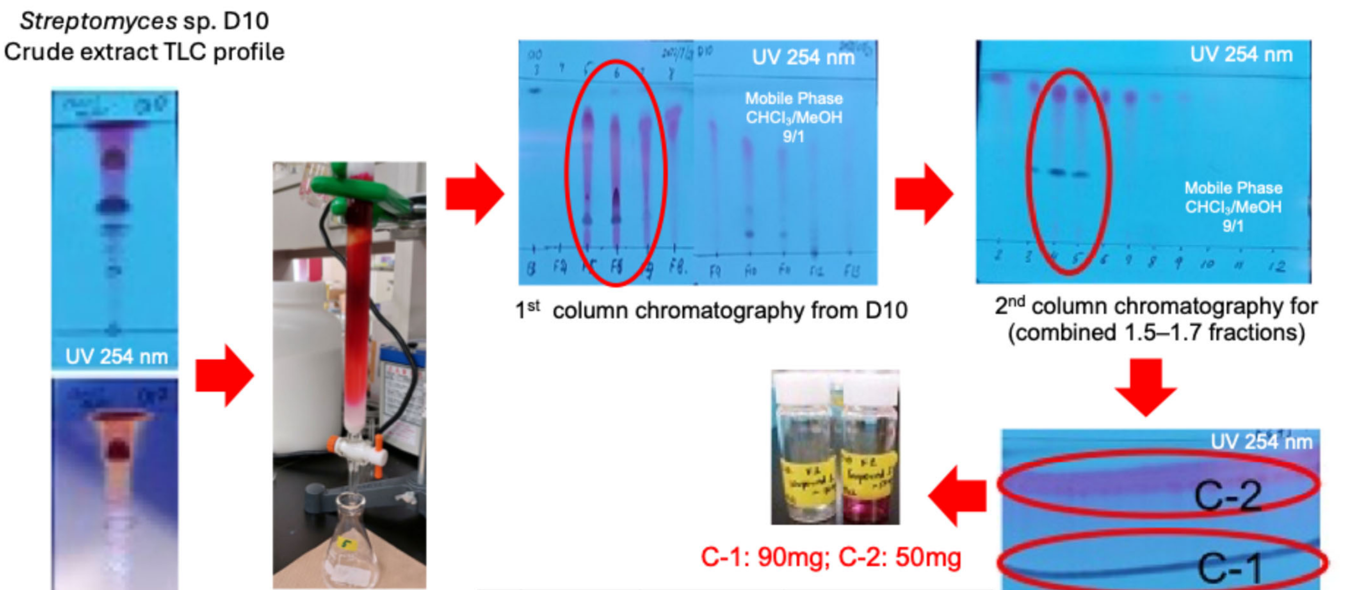

	Crude extract	Purified samples	IC_{50} *P. falciparum* 3D7
1	D10	C-1	23.80 μg/ml
2		C-2	6.55 μg/ml

Figure S2. Bioassay-guided fractionation of the crude extract from *Streptomyces* sp. D10. The crude extract was subjected to column chromatography, and fractions were monitored by thin-layer chromatography (TLC) under UV light at 254 nm and 365 nm. Fractions showing similar TLC profiles were pooled and further separated using a $CHCl_3$/MeOH (9:1) solvent system. Subsequent TLC analysis allowed separation into two major fractions (C-1 and C-2), which were isolated and collected for further analysis. The yields of the fractions were 90 mg (C-1) and 50 mg (C-2).

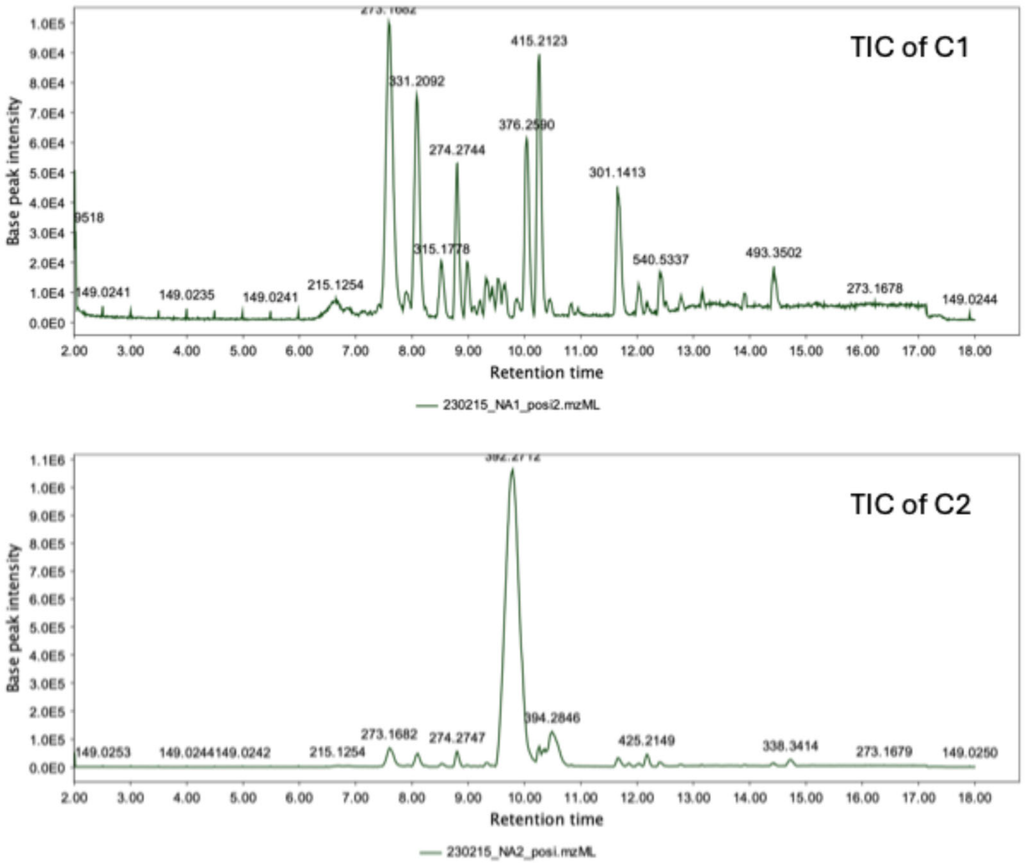

Figure S3. LC–HRMS total ion chromatograms (TIC) of fractions C1 and C2. Top panel: TIC of fraction C1 showing a complex chemical profile with multiple peaks, indicating the presence of diverse metabolites. Bottom panel: TIC of fraction C2 showing a simplified profile dominated by a single major peak, suggesting enrichment of a predominant compound after fractionation. Therefore, fraction C2 was selected for further chemical annotation and biological evaluation.

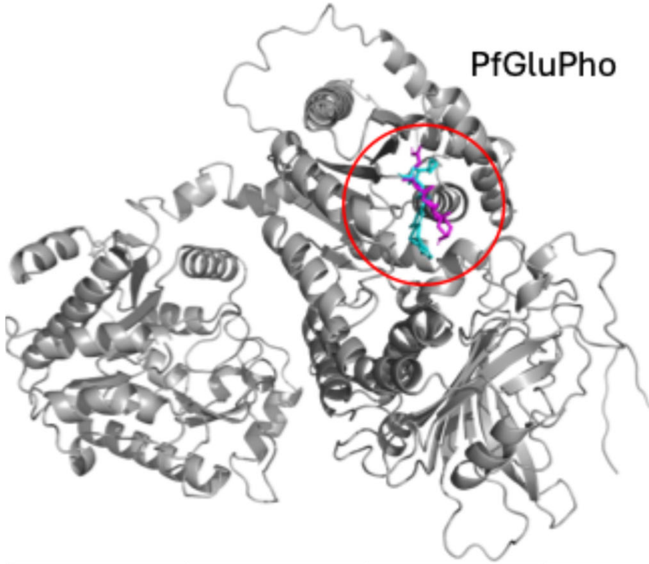

Compound	Affinity kcal/mol	CNN
UDCA	-8.04	0.9450
SBI-0797750	−8.69	0.5436

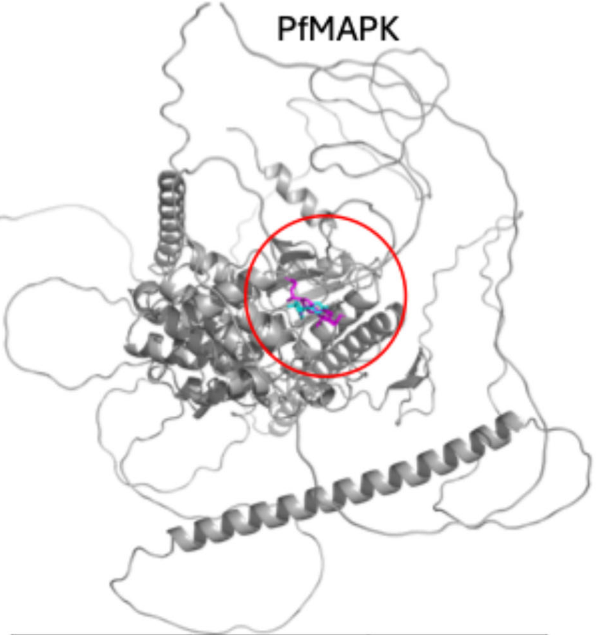

Compound	Affinity kcal/mol	CNN
UDCA	-5.46	0.8251
SB203580	−5.92	0.3207

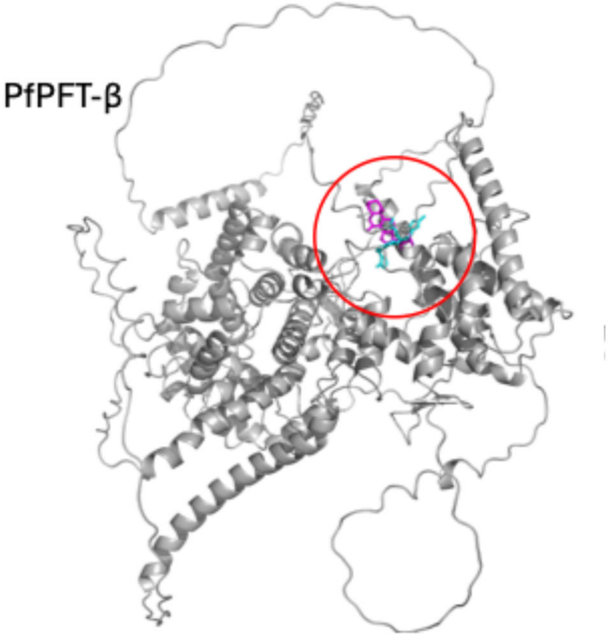

Compound	Affinity kcal/mol	CNN
UDCA	-6.53	0.8384
BMS-386914	−7.01	0.6953

Figure S4. Binding modes and predicted receptor–ligand interaction of UDCA and respective reference control for each receptor. The atom colors are defined as follows: receptor in gray, UDCA in magenta, and reference compound in cyan.
